# Trends in the healthiness and nutrient composition of packaged products sold by major food and beverage companies in New Zealand 2015 to 2019

**DOI:** 10.1186/s12916-024-03567-w

**Published:** 2024-09-11

**Authors:** Leanne Young, Bruce Kidd, Stephanie Shen, Yannan Jiang, Helen Eyles, Josephine Marshall, Sally Schultz, Jasmine Chan, Gary Sacks, Cliona Ni Mhurchu

**Affiliations:** 1 Department of Epidemiology and Biostatistics, Faculty of Medical and Health Sciences, The University of Auckland, 28 Park Avenue, Grafton, Auckland, 1023 New Zealand; 2 National Institute for Health Innovation, School of Population Health, The University of Auckland, 28 Park Avenue, Grafton, Auckland 1023 New Zealand; 3 Department of Statistics, Faculty of Science, The University of Auckland, Auckland, New Zealand; 4 The Centre for Translational Health Research: Informing Policy and Practice, School of Population Health, Faculty of Medical and Health Sciences, The University of Auckland, Auckland, New Zealand; 5 Global Centre for Preventive Health and Nutrition (GLOBE), Institute for Health Transformation, Deakin University, Geelong, VIC Australia; 6 The George Institute for Global Health, Sydney, Australia

**Keywords:** Health Star Rating, Nutrient composition, Packaged foods, Food industry, Formulation, Sodium, Sugar, Reformulation targets

## Abstract

**Background:**

Dietary risk factors are the leading cause of death globally and in New Zealand (NZ). Processed packaged foods are prevalent in the food supply and contribute excess amounts of sodium, saturated fat, and sugar in diets. Improving the nutritional quality of these foods has the potential to reduce population chronic disease risk. We aimed to evaluate the healthiness using the Australasian Health Star Rating (HSR, from 0.5 to 5 stars, with 5 being the healthiest) and nutrient composition (sodium, saturated fat, and total sugar) of packaged products manufactured by the largest NZ-based food and beverage companies in NZ 2015–2019. This analysis relates to a larger study evaluating structured engagement with food companies to improve nutrition-related policies and actions.

**Methods:**

Data was sourced from Nutritrack, a NZ-branded supermarket-sourced food composition database. The largest NZ-based companies from annual retail sales revenue (*n* = 35) were identified using 2019 Euromonitor data. All relevant products of the selected companies were extracted for analysis. Products included totalled 17,795 with a yearly range of 3462–3672 products. The primary outcome was a nutrient profile score estimated using HSR. Healthiness was defined as ≥ 3.5 stars. Secondary outcomes were sodium, total sugar, and saturated fat per 100 g/100 mL. All outcomes were assessed overall, by food company, and food category. Change over time was tested using linear mixed models, adjusting for major food categories and cluster effects of food companies controlling for multiple comparisons. Model-adjusted mean differences between years were estimated with 95% confidence intervals.

**Results:**

There was a small statistically significant increase in mean HSR between 2015 and 2019 (0.08 [0.15,0.01], *p* = 0.024). Mean total sugar content decreased over the same period (0.78 g/100 g [0.08,1.47], *p* = 0.020), but there were no significant changes in mean sodium or saturated fat contents. Seven of the 13 categories showed small increases in mean HSR (0.1–0.2). Most categories (9/13) exhibited a reduction in mean total sugar content.

**Conclusions:**

Between 2015 and 2019, there were slight improvements in the nutritional quality of selected packaged foods and drinks in NZ. Much more substantive changes are needed to address the health-related burden of unhealthy diets, supported by stronger government action and less reliance on voluntary industry initiatives.

**Supplementary Information:**

The online version contains supplementary material available at 10.1186/s12916-024-03567-w.

## Background

Dietary risk factors were one of the leading causes of death globally in 2017 [1]. Similarly, unhealthy diets and excess body weight are the foremost causes of ill health in Aotearoa New Zealand (NZ) where significant inequalities exist by ethnicity and income. However, over one-third of health loss (health loss is measured in disability-adjusted life years (DALYs), one DALY represents the loss of 1 year lived in full health) in NZ is estimated to be avoidable by stronger preventive actions, such as improving the healthiness of the food supply [2]. Packaged foods, commonly high in sodium, added sugar and saturated fat [3], dominate the food supply in most high-income countries [4] and are strongly associated with the high burden of non-communicable disease [5]. In NZ, 59% of supermarket products in 2018 were classified as unhealthy (Health Star Rating [HSR] ≤ 3.5) (0.5 to 5 stars) [6]. Therefore, improving the nutritional quality of packaged food is a population-level intervention that has the potential to have a widespread effect on population chronic disease risk, as recommended by the World Health Organization (WHO) [5] and shown to be cost-effective [7]. Furthermore, this strategy does not rely on individual behaviour change and is therefore likely to be more equitable [8].

Internationally, several government-led voluntary reformulation programmes encourage the food industry to reduce the content of sodium, saturated fat, and sugar content in food products and in 2020, the WHO released sodium benchmarks to facilitate new or existing national initiatives to reduce sodium [9]. Food formulation and reformulation are a key component of the comprehensive voluntary salt reduction programme in place in the UK since 2004 [10]. Sodium reduction was observed in several food categories, which has shown modest declines in the overall sodium content of the food supply [11] and population salt intakes [12]. Similarly in Brazil, a voluntary national sodium reduction strategy including reformulation targets resulted in a lower mean sodium content (8–34%) across more than half of the food categories between 2011 and 2017 [13]. In Australia, the government initiated the Partnership Reformulation Program, involving voluntary reformulation targets developed in partnership with the food industry and public health groups [14]. The reformulation targets focus mainly on sodium in select packaged food categories and saturated fat and sugar in a smaller number of categories [15]. Monitoring data from 2020 to 2022 shows only modest decreases in sodium and saturated fat [16].

Other levers to lower levels of nutrients of concern in some foods include a mandatory Soft Drinks Industry Levy (SDIL) in the UK. This tiered system of levies was introduced in 2018 on sugary drinks containing 5–8 g sugar/100 ml with the highest levy on drinks containing more than 8 g sugar/100 ml following a 2-year grace period prior to enforcement [17]. To avoid the higher levy companies reduced the sugar content of drinks [17].

In NZ, the current government food and nutrition policy relies predominantly on voluntary initiatives. These operate across a range of food environments including nutrition labels (HSR displayed on packaged food products to identify healthier choices within food categories), food composition (Heart Foundation-led reformulation programme which include sodium, sugar and saturated fat targets for foods [18]), food marketing (an industry-led code that limits advertisements of ‘occasional’ foods [identified by a food and beverage classification system] to children (< 14 years) [19]), and food provision in public sector education settings (Healthy Food and Drink Guidance for schools and early childhood services [20]) and the National Healthy Food & Drink policy for hospital and workplace-based food outlets [21]. In NZ, the government-funded food reformulation programme, managed by the Heart Foundation is aimed at high-volume, low-cost foods and has reported sodium reduction in key categories through incremental adjustments to nutrient criteria for sodium, and sugar over time [18, 22]. The Australasian HSR voluntary front-of-pack nutrition labelling scheme introduced in 2014 is also prompting minimal reformulation of some packaged foods; however, HSR is more likely to be selectively displayed on products with a higher HSR [23]. Between 2013 and 2019, greater reformulation was shown for packaged products in both Australia and NZ that adopted HSR compared to non-HSR-labelled products [24]. Additionally, HSR-labelled products in NZ were lower in sodium and sugar compared to non-HSR-labelled products (− 4.0% and − 2.3%, respectively) [24]. However, display of HSR on a greater number of packaged foods (24% in 2021 [23]) is necessary to increase the potential to improve population diets over time.

In 2018, the NZ government’s Ministers of Health and Food Safety requested the food industry develop an action plan to curb obesity. Subsequently a report, ‘Food Industry Taskforce on Addressing Factors Contributing to Obesity’, was produced by industry [25]. The government response to the report, in the form of a letter, recommended that the food industry take future action on reformulation, and nutrition labelling and reduce the exposure of unhealthy food and beverage marketing to children, in line with WHO recommendations [26]. However, in the intervening 5 years there has been no subsequent reporting or monitoring of food industry actions or any government monitoring of the nutritional composition of the food supply and limited strengthening of current voluntary food policy across food environments [27]. In this context, this paper aimed to evaluate the healthiness and nutrient composition of packaged foods and drinks manufactured by the largest food and beverage companies based in New Zealand over time (2015–2019).

## Methods

### Study design

Time trend analysis of the HSR (overall healthiness) and specific nutrient content (sodium, saturated fat, and total sugar) of packaged food and non-alcoholic beverages available in NZ supermarkets, by major NZ-based food company and food category, from 2015 and 2019.

### Data source

Nutrient composition data for packaged foods were obtained from Nutritrack (years 2015 to 2019 inclusive), a branded database of packaged foods and beverages sold at NZ supermarkets comprising ~ 15,000 foods/year [28]. The data are collected from four major supermarkets (New World, 4Square, Countdown and PAK’nSAVE) each year (March–May) in the largest NZ city (Auckland) and represent the two dominant national supermarket retailers (Woolworths NZ owns Countdown and Foodstuffs NZ owns New World, 4Square and PAK’nSAVE). Together, Woolworths NZ and Foodstuffs NZ account for 90% of the grocery market share, nationally in 2022 [29]. Data collected for each product includes the barcode, product name, brand name, packet size, serve size, nutrient information panel (NIP) data, ingredients, and whether the product displays a Health Star Rating (HSR) label on packaging. Products are categorised into food groups and smaller categories using an amended version of the Global Food Monitoring Food Classification system [30] which include five levels of categorisation.

### Sample of food companies

Companies were included if they produced packaged food and/or non-alcoholic beverages, had an annual retail sales revenue of > $10 million NZD (> 6 million USD) per annum (2019), operated a holding Company or Head Office and manufacturing site located in New Zealand, and had a portfolio of at least 10 products considered amenable to reformulation. Euromonitor data (2019) was used to obtain annual retail sales revenue (and market share) for each company, disaggregated by Euromonitor’s ‘packaged food’ and ‘soft drinks’ categories. ‘Packaged food’ included all baked, dried, and chilled packaged foods sold through establishments primarily engaged in the sale of fresh, packaged and prepared foods for home preparation and consumption (e.g. dairy, snack foods, canned or frozen fruit and vegetables, snack food, confectionary, cereal products, processed meat and seafood, sauces and spreads). ‘Soft drinks’ included non-alcoholic beverages (e.g. carbonates, fruit/vegetable juice, bottled water, functional drinks, concentrates, RTD (ready to drink) tea, RTD coffee and Asian speciality drinks) [31]. Companies excluded were those that manufactured chewing gum products, sports supplements, or infant formula only, as were supermarkets, retailers, distributors, importers, or wholesalers only, and companies whose product nutrition information was not available in the Nutritrack database and therefore nutritional profile and HSR was unable to be determined. This analysis was part of a larger randomised trial evaluating structured engagement with food companies as a method to improve nutrition-related policies and actions [32].

### Data preparation

For each year, all relevant products were selected across 12 major Nutritrack food categories: Bread and Bakery Products, Cereals and Cereal Products, Confectionery, Convenience Foods, Dairy, Edible Oils and Emulsions, Fish and Seafood Products, Fruit and Vegetables, Meat and Meat Products, Non-alcoholic Beverages, Sauces and Spreads, and Snackfoods.. The remaining four food categories (Eggs, Sugars, Honey, and Related Products, Vitamins and Supplements and Special Foods) were excluded as products were not eligible for HSR or amenable to reformulation. Products were matched to their corresponding food company using publicly available information on food company websites (brands, product lists and new brand acquisitions), news reports of brand ownership changes (through internet searches), and product packaging photographs in the Nutritrack database. Brand ownership changes were assigned 1 year after the year documented in public media/news reporting to allow for product packaging changes. Products produced by a company and distributed by another company were linked to the company manufacturing the product.

**Fig. 1:**
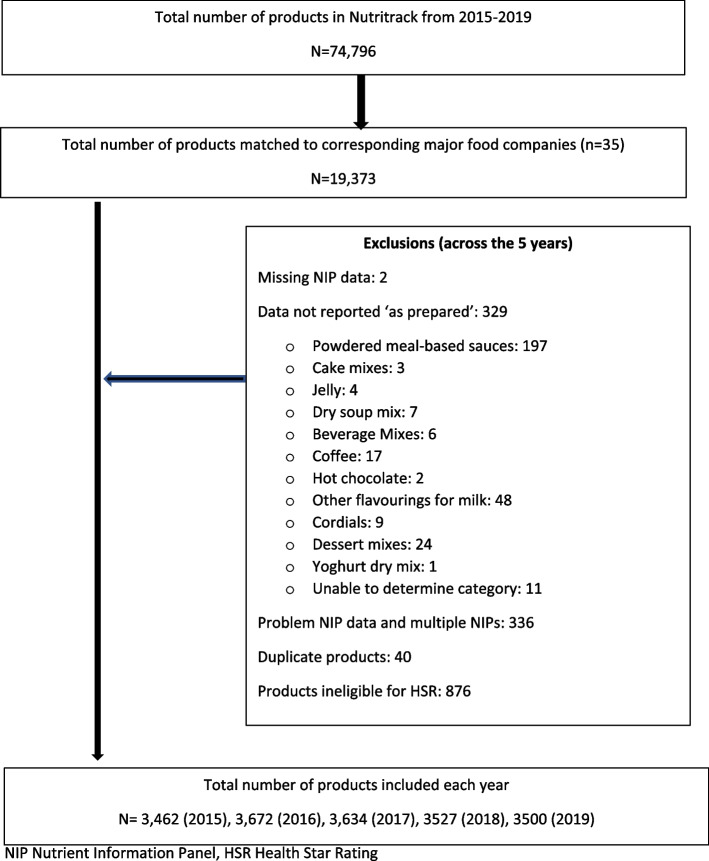
Flow chart of data preparation and the number of products included in the trend analysis of HSR and nutrient content 2015–2019

Products were excluded from analyses if (i) there was missing NIP data required for the calculation of HSR (energy, saturated fat, total sugar, sodium or protein content) or for the particular nutrient content (sodium, total sugar, saturated fat) (e.g., if a product was missing sodium content it would be excluded from the calculation of HSR and sodium only) (*N* = 2); (ii) the product nutrient data were not reported ‘as prepared’ (this was assessed in the following categories that can be reconstituted; meal-based sauces, gravies and stocks, meal accompaniment sauces, cake mixes, jelly, dry soup mixes, beverage mixes, cordials, dessert mixes, yoghurt dry mix, diet soup and drink mixes (*N* = 329); (iii) products displaying obvious errors on the NIP (e.g. carbohydrate less than total sugar) or which had multiple NIPs e.g., variety packs (*N* = 336), iv) duplicate products (*N* = 40); and (v) products ineligible for the Health Star Rating (baby foods, meal kits, diet soup mixes [meal replacements], diet drink mixes, chewing gum, cough lollies, herbs and spices, salt, tea, vinegars, protein powders, sports gels and protein and diet bars) (*N* = 876) (Fig. 1). Products with US NIPs where nutrient data is displayed per serve, were converted to per 100 g or per 100 ml.

### Outcome measures

The primary outcome was the HSR score calculated using the 2021 version of the algorithm [33]. HSR is a nutrient profile model that uses star ratings, 0.5 to 5 stars, to indicate the healthiness of packaged foods [34]. The rating is based on the energy, saturated fat, sodium, and sugar content and additionally, fibre, protein, fruit, vegetable, nut and legume (FVNL) content of the food. It is designed to provide consumers with information to indicate the relative healthiness of foods within food categories and guide informed choices [34]. As the use of the HSR by food companies in NZ is limited [23], an algorithm in the Nutritrack database estimates the score for each product. Estimation of HSR in Nutritrack has been previously described [35], and involves the categorisation of products according to the HSR food categorisation system, use of the NIP data and the published HSR algorithm with estimations for the fibre and fruit, vegetable, nut and legume (which are not mandatory on NIPs in NZ) content of the food to determine the number of stars a product is eligible to display [33]. The algorithm is not used for the following categories which instead are eligible for an automatic HSR of five depending on their ingredients: fresh and minimally processed fruit and vegetables (includes fresh and frozen, excludes dried and canned in brine or juice); sparkling water; still water; and an automatic HSR of 4.5 for other unsweetened flavoured waters. Healthy products were defined as those equal to or above 3.5 stars (out of a maximum of 5), in line with other similar studies [6, 36]. The cut-off of ≥ 3.5 has shown reasonable capability to identify healthier foods aligned with the Nutrient Profiling Scoring Criterion, a scoring system used to determine eligibility to display health claims on foods in NZ [37]. Secondary outcomes were sodium (mg per 100 g or 100 mL), total sugar (g per 100 g or 100 mL) and saturated fat (g per 100 g or 100 mL) contents.

### Statistical analysis

All eligible products from company product portfolios were included in each analysis. The mean (SD) HSR score and nutrient values per 100 g or 100 mL were calculated and summarised for each company per year, overall and by major food categories. Change over time was tested using linear mixed models, adjusting for major food categories and the cluster effect of food companies. The fixed effect model included both year and major food category as categorical explanatory variables, as we did not believe that the change in HSR and nutrient outcomes showed a linear trend over time. The random effect model included food company as a cluster effect, with the assumption that the products produced by same food companies would be more similar (correlated) than those produced by different companies, The model estimated both within-cluster and between-cluster variances separately. Since our product database is large (17,952 products in total, ~ 3500 products per year), the mean outcome measures are approximately normally distributed based on Central Limit Theorem. Model-adjusted means and the differences between individual years e.g., 2015 and 2019 were estimated with 95% confidence intervals. All statistical tests were adjusted for multiple comparisons using the Tukey–Kramer test and maintained at a 5% significance level. Statistical analysis was performed using SAS version 9.4 (SAS Institute Inc., Cary, NC, USA).

## Results

### Sample

The analysis included the largest NZ-based food and beverage companies (*n* = 35). The market share (2019) (retail value) and annual category sales revenue by company as well as the number of brands and products included in the analysis from each company overall and by year, are shown in Additional file 1: Table S1. The total number of products included in this analysis for each of the 5 years was 3462 in 2015, 3672 in 2016, 3634 in 2017, 3527 in 2018 and 3500 in 2019. Food categories with the largest numbers of products overall were dairy (*n* = 2819, 15.9%) and non-alcoholic beverages (*n* = 2671, 13.4%). The market share (retail value) of NZ-based companies in the sample ranged from 0.1 to 12% for the packaged food category and from 3 to 41% for the non-alcoholic beverages category (Additional file 1: Table S1).

### Healthiness and nutrient content

#### Total 2015–2019

**Fig. 2:**
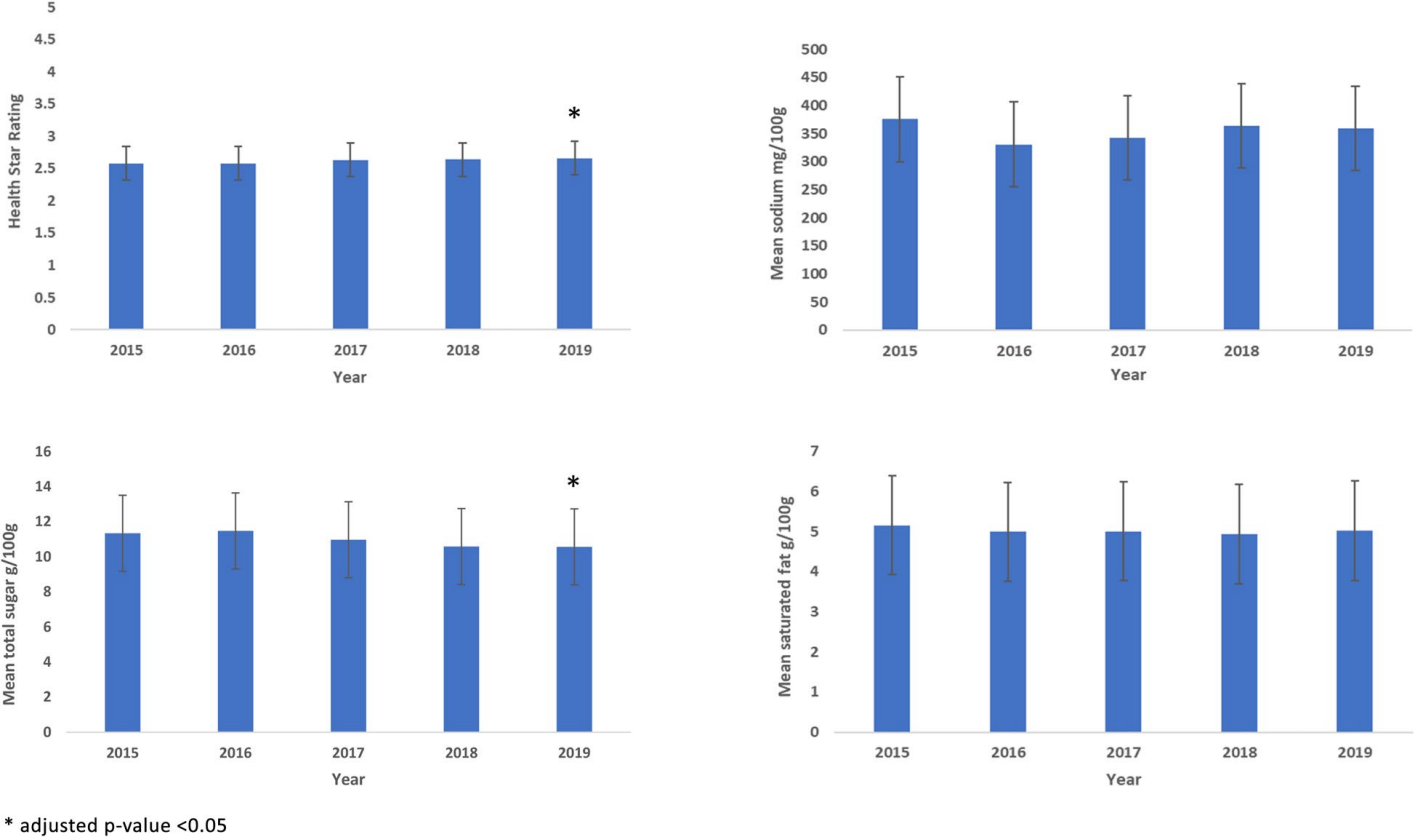
Mean (CI) HSR, sodium, total sugar and saturated fat content of packaged foods from 35 New Zealand-based food companies 2015–2019

The mean HSR, sodium (mg/100 g), total sugar (g/100 g), and saturated fat (g/100 g) of all products each year from 2015 to 2019 are shown in Fig. 2. The mean HSR in 2019 and for all products examined (*N* = 17,681) was 2.70. The mean sodium, total sugar, and saturated fat content in 2019 were 356.1 mg/100 g, 11.2 g/100 g, 4.8 g/100 g, respectively.

Compared to 2015, there was a small significant increase in mean HSR in 2019 (0.08 [0.15, 0.01], *p* = 0.024) (3% increase) (Fig. 2). For total sugar, there was a small significant reduction in 2019 (0.78 g/100 g [0.08, 1.47], *p* = 0.020) (7% decrease). There were no significant differences in saturated fat (*p*=0.82) and sodium content (*p*=0.85) between 2015 and 2019. Estimated mean differences for HSR, total sugar, sodium and saturated fat between years are shown in Table 1.

**Table 1:** Estimated mean differences in Health Star Rating, total sugar, sodium, and saturated fat content of packaged food products from major New Zealand-based food companies between years with adjustment for multiple comparisons

Year	Vs year	Mean difference in HSR (95% CI)	Mean difference in sodium (mg/100 g or mg/100 mL) (95% CI)	Mean difference in total sugar (g/100 g or g/100 mL) (95% CI)	Mean difference in saturated fat (g/100 g or g/100 mL) (95% CI)
2015	2016	0.001 (− 0.069, 0.072)	45.378 (1.711, 89.046)*	− 0.134 (− 0.820, 0.553)	0.167(− 0.168, 0.502)
2015	2017	0.050 (− 0.121, 0.020)	33.924 (− 9.827, 77.673)	0.371 (− 0.317, 1.058)	0.150 (− 0.186, 0.486)
2015	2018	0.055 (− 0.126, 0.016)	12.409 (− 31.667, 56.485)	0.756 (0.063, 1.449)*	0.217 (− 0.122, 0.555)
2015	2019	0.078 (− 0.149, − 0.007)*	16.487 (− 27.719, 60.693)	0.776 (0.081, 1.471)*	0.135 (− 0.205, 0.474)
2016	2017	0.052 (− 0.121, 0.017)	− 11.454 (− 53.315, 31.406)	0.504 (− 0.170, 1.178)	− 0.017 (− 0.346, 0.312)
2016	2018	0.056 (− 0.126, 0.013)	− 32.970 (− 76.163, 10.224)	0.889 (0.210, 1.569)*	0.050(− 0.282, 0.382)
2016	2019	0.079 (− 0.149, − 0.009)*	− 28.891 (− 72.231, 14.489)	0.910 (0.228, 1.591)*	− 0.032 (− 0.365, 0.301)
2017	2018	− 0.005 (− 0.074, 0.065)	− 21.515 (− 64.734, 21.704)	0.385 (− 0.295, 1.065)	0.067 (− 0.265, 0.400)
2017	2019	− 0.028 (− 0.097, 0.042)	− 17.437(− 60.771, 25.897)	0.406 (− 0.276, 1.087)	− 0.015 (− 0.348, 0.318)
2018	2019	− 0.023 (− 0.093, 0.047)	4.078 (− 39.581, 47.737)	0.021 (− 0.666, 0.707)	− 0.082 (− 0.418, 0.253)

Model-adjusted mean differences and 95% confidence intervals were reported with adjustment for multiple comparisons using the Tukey–Kramer test

^*^ adjusted *p*-value < 0.05

#### Change by company

Changes in HSR, sodium, total sugar and saturated fat contents varied by company, category and nutrient (Table 2).

**Table 2:** Mean Health Star Rating, sodium, total sugar, and saturated fat between 2015 and 2019 by company

NZ companies *N*=35	Health Star Rating				Sodium mg/100g				Total sugar g/100g				Saturated fat g/100g			
	2015		2019		2015		2019		2015		2019		2015		2019	
	N	Mean	N	Mean	N	Mean	N	Mean	N	Mean	N	Mean	N	Mean	N	Mean
1	370	2.8	320	2.9	373	310.6	320	318.7	371	9.9	320	8.7	373	11.0	320	11.3
2	731	3.0	622	3.0	731	607.0	625	462.4	737	10.9	622	9.7	735	1.4	622	1.3
3	141	1.5	179	1.8	144	428.2	179	446.6	142	19.2	179	18.5	143	9.2	179	7.8
4	193	2.3	203	2.4	194	405.1	203	297.4	196	16	203	20.4	199	5.2	203	5.1
5	52	1.5	79	1.5	52	656.1	79	658.1	52	5.3	79	4.0	52	11.4	79	8.6
6	96	1.7	101	1.7	97	910.2	101	933.7	96	1.6	101	1.7	97	6.0	101	5.5
7	55	3.7	62	3.9	55	362.7	62	238.5	55	10.1	62	10.8	55	1.4	62	1.5
8	62	4.1	58	3.8	62	181.1	58	257.9	62	3.5	58	3.5	62	1.5	58	1.8
9	116	3.8	85	3.8	117	368.2	85	378.8	120	2.3	85	2.3	119	1.4	85	1.4
10	49	0.5	69	0.5	51	64.6	69	67.0	51	46.3	69	43.9	49	17.1	69	18.3
11	59	3.1	54	3.1	59	614.5	54	618	59	1.3	54	1.3	59	18.5	54	18.3
12	31	3.8	18	3.5	31	303.7	18	398.2	31	4.1	18	3.6	31	1.2	18	1.8
13	73	3.6	76	3.6	76	440.8	76	474.7	74	1.6	76	1.8	76	2.3	76	2.1
14	128	2.7	118	3.0	128	180.4	118	164.3	128	22.6	118	19.8	128	7.8	118	7.9
15	67	3.1	85	3.3	67	94.6	85	88.3	67	24.2	85	29.9	67	7.9	85	6.9
16	25	1.3	42	1.4	25	106.3	42	116.5	25	55.1	42	45.3	25	3.3	42	4.4
17	141	2.7	118	2.9	141	648.4	118	690.3	141	9.0	118	7.5	141	2.4	118	2.6
18	69	3.1	81	3.8	69	503.5	81	445.9	69	4.4	81	4.2	69	3.3	81	2.1
19	39	0.7	34	0.7	39	1456.5	34	1393.1	39	0.9	34	1.2	39	9.9	34	8.2
20	28	1.5	39	1.4	28	1074.4	39	1129	28	0.9	39	0.9	28	5.7	39	6.1
21	18	3.4	11	3.4	18	216.9	11	224.3	18	1.7	11	1.9	18	1.3	11	1.0
22	37	1.6	44	1.6	37	44.9	44	48.8	37	23.0	44	22.6	37	9.2	44	8.6
23	34	3.7	19	3.2	34	257.4	19	124.8	36	11.1	19	14.8	36	1.8	19	5.0
24	36	3.4	50	3.8	36	98.3	50	58.3	36	11.3	50	9.0	36	3.5	50	2.5
25	23	1.8	15	2.0	23	54.0	15	59.0	23	22.0	15	21.2	23	7.6	15	6.8
26	28	3.2	29	3.7	29	579.7	29	410.0	29	10.7	29	2.2	29	2.6	29	2.2
27	14	0.8	35	1.2	15	298.1	35	259.6	15	34.9	35	34.5	15	14.1	35	12.6
28	11	3.3	36	2.1	11	77.9	36	144.2	11	4.2	36	8.1	11	11.1	36	13.0
29	11	3.0	17	2.6	11	395.5	17	536.4	11	10.6	17	10.1	11	3.5	17	4.3
30	155	3.7	208	3.7	158	125.1	208	380.5	158	9.3	208	8.4	159	6.3	208	5.0
31	83	3.3	87	3.3	84	442.2	87	455.1	85	3.9	87	3.9	84	2.7	87	2.5
32	20	1.8	25	2.0	20	780.3	25	733.7	20	1.2	25	1.1	20	5.0	25	4.7
33	167	1.7	245	1.9	165	14.4	245	16.2	162	7.3	243	6.5	166	0.0	243	0.0
34	157	2.3	186	2.0	150	11.4	186	23.3	149	9.1	184	7.9	149	0.2	184	0.2
35	66	2.1	55	2.0	64	6.3	55	3.4	64	8.8	55	8.2	64	0.1	55	0.0

#### Change by category

Between 2015 and 2019 seven of the 13 categories showed small increases in mean HSR (Table 3). Around half (6/13) the categories recorded a small decline in mean sodium content. Most categories showed a small reduction in mean total sugar content and only three categories showed a small reduction in mean saturated fat.

**Table 3:** Mean HSR, sodium, total sugar, and saturated fat content between 2015 and 2019 by food category

Food and beverage categories	Health Star Rating				Sodium mg/100g				Total sugar g/100g				Saturated fat g/100g			
	2015		2019		2015		2019		2015		2019		2015		2019	
	N	Mean	N	Mean	N	Mean	N	Mean	N	Mean	N	Mean	N	Mean	N	Mean
Bread and bakery products	196	2.4	235	2.2	199	397.6	235	457.3	199	15.4	235	14.4	199	5.7	235	5.9
Cereal and cereal products	308	3.2	338	3.4	309	194.3	338	160.2	310	15.3	338	13.5	311	3.1	338	3.2
Confectionery	152	0.8	201	0.9	154	67.3	201	78.1	155	48.0	201	45.5	154	11.9	201	13.0
Convenience foods	234	3.4	194	3.5	235	288.7	194	287.6	238	2.8	194	2.8	238	1.2	194	1.3
Dairy	555	2.9	524	3.0	558	265.9	524	250.3	556	10.7	524	10.1	558	9.3	524	9.0
Edible oils and oil emulsions	33	1.9	33	1.6	33	251.4	33	291.0	33	0.8	33	0.7	33	41.8	33	43.4
Fish and seafood products	129	3.5	108	3.4	129	474.2	108	509.6	132	2.0	108	1.9	131	1.9	108	2.2
Fruit and vegetables	476	3.9	466	3.9	480	450.8	466	301.8	482	11.6	466	12.3	481	2.2	466	2.6
Meat and meat products	332	2.3	333	2.4	337	777.9	333	803.9	335	1.7	333	1.7	338	5.1	333	4.7
Non-alcoholic beverages	442	2.1	564	2.1	431	13.6	564	20.3	427	8.1	560	7.2	431	0.3	560	0.3
Sauces and spreads	382	2.3	364	2.5	382	869.8	364	853.2	384	11.9	364	11.1	385	2.4	364	2.4
Snackfoods	139	1.4	136	1.6	140	674.9	136	686.3	139	3.8	136	3.3	139	10.8	136	8.4

## Discussion

### Summary of findings

This study of a subset of the NZ food supply, observed a small, statistically significant increase in mean product nutrient profile (HSR score). It is possible, but not certain, that this was due to the concomitant small, statistically significant, decrease in mean total sugar content over the same period; it could also have been due to changes in other nutrients and components considered in the HSR. Mean saturated fat content did not change and there was no consistent reduction in mean sodium content during the study 5-year time frame. A company producing packaged food from the Meat alternatives and Sauces and Spreads categories showed the largest increase in mean product HSR and around half of the categories recorded small increases in mean product HSR between 2015 and 2019. Overall, the observed changes in the composition of packaged foods from a sample of NZ-based food companies were minimal over this period.

Possible reasons for the observed change in HSR include reformulation, introduction of new (healthier) products [38], removal of existing (less healthy) products, and/or changes in brand ownership which would change the overall company product portfolio.

### Previous NZ and international research on the overall healthiness of supermarket food supply

Our finding of an overall mean (SD) HSR of 2.70 across all products and years is the same as a larger previous NZ cross-sectional study in 2018 (*N* = 13,506 products) that reported an overall mean HSR of 2.70 [6]. Our results generally align with similar studies in the US (HSR 2.70) (*N* = 230,156) [39] and Australia (HSR 2.80) (*N* = 40,664) [40] although the studies are not directly comparable due to differences in methodology including product sampling methods. Additionally, an intercountry study reported a comparable overall mean HSR of 2.73 (SD 1.38) for packaged foods across all 12 countries (*N* = 394,815) and a country-specific mean HSR for NZ of 2.73 (*N* = 19,383) [36].

### NZ policies and programmes to improve the food supply

Minimal improvement in the nutrient profile of packaged food found in this study is a public health concern given that New Zealanders have high intakes of sodium [41], total sugar [42], and saturated fat [43]. The nutritional quality of the food supply, specifically nutrients of concern (sodium, saturated fat, added sugar content of foods), has been shown to be an important driver of chronic disease [5]. NZ has a range of government-led voluntary food and nutrition policies, and food industry-led codes and programmes that provide guidance on the nutrient composition of foods in a range of settings and environments. These include government policies the Health Star Rating, the National Healthy Food & Drink policy in hospitals and workplaces [21], and the Healthy Food and Drink Guidance for schools and early childhood education services [20]. Industry-targeted programmes include the Heart Foundation-led reformulation programme [22], and the Advertising Standards Authority Children and Young Person’s Advertising Code [19]. However, there is little evidence that these voluntary policies, codes, and programmes have led to substantive improvements in the healthiness of the packaged food supply [22, 23] or reduced exposure of children to marketing of unhealthy foods and brands. Internationally, NZ scored a ‘2’ out of a possible ‘4’ for implementation of recommended actions on sodium reduction by the WHO [44]. In contrast, government-led reformulation programmes in other countries have shown a positive impact on food composition including the sodium reduction programme in the UK [10] and Brazil [13], the soft drink industry levy on sugar content in the UK [17], warning labels on products high in sodium and sugar in Chile [45], and mandatory sodium targets in South Africa [46] and Argentina [47]. In this context, the results of this study reinforce that limited implementation of government-led policies in NZ and a heavy reliance on weak voluntary industry initiatives means there has been no meaningful improvements in the healthiness of the food supply.

### Voluntary actions to improve the food supply

Voluntary actions to improve the food supply are often preferred by governments and the food industry. For governments this is because of the complexity and high set-up and enforcement costs of regulation and strong opposition from individual food companies or industry bodies and the food industry want to forestall mandatory or more restrictive government requirements [48]. The lack of strength (breadth and scope) of voluntary codes has been identified as a barrier to the effectiveness of voluntary actions to improve public health nutrition. For example, the Australian voluntary reformulation program has been found to have lenient targets and apply only to a subset of product categories [49]. In addition, industry self-regulatory codes on food marketing typically do not meet WHO recommendations and have been consistently found to be ineffective at reducing the exposure of children to unhealthy food advertising [50], although the food industry often reports good adherence [51]. High food industry involvement in food policy development has been shown to limit success in improving population nutrition outcomes [52]. While some involvement of the food industry when initiating food policy is necessary, high participation and oversight by the government is recommended over industry influence [52]. Furthermore, voluntary actions lack structured monitoring, reporting of compliance or performance, thus there is little accountability on the part of the food industry [48]. The outcomes of voluntary participation in the HSR scheme in NZ since 2014 show HSR is only displayed on 24% of products and a small effect on reformulation (sodium [− 4.0%], and sugar [− 2.3%]) [23]. It is hindered by the selective display of HSR labels on already healthier products despite reformulation effects being greater when applied to less healthy products [23]. Newly introduced government uptake targets for HSR in December 2020, following the 5-year review of the scheme (50% of intended products by November 2023, 60% by 2024 and 75% by 2025), aim to increase industry participation and thus reformulation and formulation [53]. These were introduced despite strong calls from public health and consumer advocacy groups to make the HSR scheme mandatory [54].

### Food reformulation/formulation programmes to improve the food supply

Food reformulation programmes, that specify maximum levels of sodium for foods, have been shown to incentivise industry reformulation and reduce population intake [10], yet NZ has not implemented a government-led food reformulation programme. This is despite public experts identifying this as a priority action (among other recommendations) to improve the healthiness of food environments since 2011 [55]. The NZ State of the Food Supply report shows wide ranges of nutrient contents within categories signalling opportunities for reformulation and healthier nutrient formulations [6]. Assessment of NZ food industry actions indicated some progress on reformulation, specifically sodium reduction, and mainly by retailers [56]. Vandevijvere S, Kasture A [56] called for the industry to extend the scope of reformulation to all risk nutrients and across all companies and products, as well as to adopt time-bound, measurable reformulation targets and public reporting of progress. The results of our study support these findings and previous research that highlight the persistent lack of action from food companies in NZ [56, 57]. Although voluntary, important features of the original UK sodium reduction programme were government leadership with strong promotion and encouragement to achieve voluntary targets and comprehensive monitoring by the government, combined with the caveat of mandatory targets if action on reformulation was insufficient. Findings show that between 2003 and 2011, there was a 20% or higher drop in the mean sodium content in many key food categories and a concomitant reduction in average adult salt intake (9.5 to 8.1 g/day) [12, 58]. In contrast, Argentina introduced mandatory targets in 2013 allowing the industry 1 year to comply, and an independent survey in 2017–2018 showed just 6% of products exceeded the targets [47]. South Africa also introduced mandatory targets (2016) and 67% of all products met the mandated targets in the initial phase of the programme in 2016 [46]. These studies indicate that the food industry may be more likely to improve nutrient composition in the face of strong government-led policy incorporating mandated or regulated targets.

Our findings show a need for policy and actions to improve the healthiness and nutrient composition of the food supply in NZ. This should include a government-led reformulation programme with nutrient targets set for all major food categories. Mandatory regulations are likely to have the greatest impact on public health. In the absences of regulation, a voluntary, government-led programme could be implemented if it is supported by a comprehensive monitoring and evaluation plan, with the ability to progress to a more regulatory approach if food industry actions are limited.

### Strengths and limitations

This repeated cross-sectional analysis measured food composition over 5 years to assess changes in nutrient levels and included over 3000 packaged products each year (2015–2019) from 35 major food and drink companies operating in NZ. A strength was that it utilised a large, well-established packaged food composition database with good quality control processes [28]. Based on nationally representative market research panel data, the Nutritrack database includes ~ 75% of all food purchases made by NZ households [28]. Companies with head offices and/or manufacturing facilities in NZ may be more able to change local product formulations compared to companies with head offices or manufacturing facilities based overseas. Furthermore, this study used HSR to assess the healthiness of foods as it is widely used for this purpose in other studies and evaluations of food supply healthiness [6, 36]. A cut-off of 3.5 stars was used to define healthy products similar to previous research in NZ [6] and overseas [36]. Use of a HSR of 4 as the cut-off is likely to have shown a less healthy food supply. However, a cut-off of 3.5 stars has been questioned due to its categorisation of some discretionary foods as healthy [59]. Future research to assess the healthiness of the NZ food supply could consider inclusion of one or more of the following; an assessment of the level of processing using NOVA [3] and comparison of the nutrient composition with relevant benchmarks such as the NZ Heart Foundation reformulation targets [18], Australian Healthy Food Partnership targets [14], UK sodium and sugar targets [10, 17] and the global WHO global sodium benchmarks [9]. Reformulation is just one approach to improving diets and there also needs to be efforts to rebalance diets to include more whole, unprocessed foods and fewer packaged foods.

The 35 companies included in this study only accounted for approximately 25% of products in the Nutritrack database each year 2015–2019, therefore focussing on this small number of companies is not representative of the broader NZ food market. Also, Nutritrack data collection may not have included all company products since collections were only completed at four major supermarket stores. However, selection criteria for this study focussed on identifying the largest companies by revenue, and thus those most likely to have the greatest influence on the packaged food supply. A previous cross-sectional study of the NZ food supply showed that over two thirds of the packaged food (67%) and drinks (77%) are supplied by only 19 and 3 companies, respectively [6]. The previous study sample differed from this sample however, in that all products in the Nutritrack database (*N* = 13,506) were matched to parent companies, not all companies were NZ-owned or based, and two major retail chains with wide ranges of private label products were included. Another limitation is that food companies may have added new products with lower sugar, sodium or saturated fat content rather than reformulate existing products [38], a strategy commonly used by food industry to improve the nutritional profile of product portfolios, unquantified in our study. This may be driven by the lower cost of formulation compared to reformulation, technological problems with reformulation and consumer preferences for existing product taste profiles [38]. Our study could have been strengthened by weighting results by sales, and a longer study time frame (> 5 years) to allow more time for product reformulation/formulation. Exclusion of retailers limited the number of eligible products.

Furthermore, the Nutritrack dataset used for the study are based on annual cross-sectional audits of products found in-store and may not have captured all available products from each company for sale in each year (data collected during one-quarter each year). Product HSRs were also based on estimations, due to the low uptake of the voluntary front-of-pack HSR labelling system by the food industry [23, 35, 60]. Estimation of HSR requires imputation of some food components when unavailable or limited information was provided in the nutrient information panel or ingredient list (e.g., fibre and fruit, vegetable, nut, and legume content), as has been reported elsewhere [35, 60] although it is possible that some product HSRs may have been over/underestimated with this approach.

## Conclusions

Packaged foods and drinks supplied by a selected sample of major companies based in NZ were generally unhealthy, scoring an average HSR of 2.7 stars out of a possible five across all products and years. Product nutrient composition has changed little over time, between 2015 and 2019. This is likely due to the predominance of voluntary nutrition-related initiatives and a lack of strong government action to address the domination of unhealthy foods in the NZ food supply. Robust government policies, coupled with strong regulatory and monitoring mechanisms are needed to substantially improve the healthiness of packaged foods and drinks in NZ.

## Supplementary Information

Additional file 1: Table S1. Characteristics of major food companies in New Zealand

## Data Availability

The Nutritrack database is owned and managed by The University of Auckland. The methods may be published if cited appropriately. Access to the data for external research collaborators has now closed. Access to the data for internal collaborators may be available—please contact the principal investigator with inquiries.

## Abbreviations

AUD: Australian dollars
HSR: Health Star Rating
NZ: New Zealand
NIP: Nutrient Information Panel
NZD: NZ dollars
RTD: Ready to drink
SDIL: Soft Drinks Industry Levy
UK: United Kingdom
USD: US dollars
WHO: World Health Organization
