# Supplementary material for: Trends in the healthiness and nutrient composition of packaged products sold by major food and beverage companies in New Zealand 2015 to 2019
Source: BMC Med. 2024 Sep 11;22:372. doi: 10.1186/s12916-024-03567-w (PMC11389062; doi:10.1186/s12916-024-03567-w)
Supplement: Supplementary file 1 — Additional file 1: Table S1. Characteristics of major food companies in New Zealand [file 12916_2024_3567_MOESM1_ESM.docx]

**Additional File 1: Table S1:** **Characteristics of major food companies in New Zealand**

| NZ companies  (N=35) | Type of products in company’sportfolio, by category | Category  annual  sales  revenue  (2019 retail value), by category | Company  market  share  (2019 retail  value), by category | Year | | | | | | | | | | | | | Total | | |
| --- | --- | --- | --- | --- | --- | --- | --- | --- | --- | --- | --- | --- | --- | --- | --- | --- | --- | --- | --- |
|  |  |  |  | 2015 | | 2016 | | 2017 | | | | 2018 | | 2019 | | | | |  |
|  |  | USD million^a^ | % | Number of brands | Number of products | Number of brands | Number of products | | Number of brands | Number of products | Number of brands | | Number of products | | Number of brands | Number of products | | Number of products | |
| Category: Packaged food | | |  |  |  |  |  | |  |  |  | |  | |  |  | |  | |
| 1 | Dairy; edible oils, snack foods | 890.6 | 12.3 | 15 | 374 | 15 | 402 | | 17 | 356 | 15 | | 333 | | 14 | 321 | | 1786 | |
| 2 | Fruit and vegetables (canned); sauces & spreads, convenience foods | 313.8 | 4.3 | 35 | 748 | 32 | 695 | | 34 | 685 | 29 | | 672 | | 29 | 622 | | 3422 | |
| 3 | Bread & bakery products; snack foods | 228.7 | 3.2 | 5 | 148 | 6 | 154 | | 7 | 139 | 6 | | 128 | | 6 | 179 | | 748 | |
| 4 | Cereal and cereal products, confectionery, sauces & spreads | 158.9 | 2.2 | 13 | 203 | 14 | 177 | | 12 | 206 | 11 | | 174 | | 7 | 203 | | 963 | |
| 5 | Snack foods | 136.6 | 1.9 | 4 | 52 | 7 | 78 | | 6 | 73 | 6 | | 76 | | 7 | 79 | | 358 | |
| 6 | Meat and meat products | 120.7 | 1.7 | 2 | 97 | 6 | 122 | | 6 | 112 | 6 | | 106 | | 5 | 101 | | 538 | |
| 7 | Cereal and cereal products; special foods (breakfast beverages) | 110.3 | 1.5 | 4 | 55 | 4 | 57 | | 4 | 58 | 4 | | 59 | | 4 | 62 | | 291 | |
| 8 | Fruit and vegetables (frozen), convenience foods; | 74.8 | 1.0 | 2 | 62 | 2 | 72 | | 2 | 68 | 2 | | 64 | | 1 | 58 | | 324 | |
| 9 | Fish and seafood products | 73.0 | 1.0 | 3 | 123 | 4 | 98 | | 3 | 101 | 3 | | 90 | | 2 | 85 | | 497 | |
| 10 | Confectionery (chocolate) | 67.7 | 0.9 | 1 | 52 | 1 | 54 | | 1 | 56 | 1 | | 60 | | 1 | 69 | | 291 | |
| 11 | Dairy, edible oils, milk | 63.3 | 0.9 | 3 | 59 | 4 | 79 | | 4 | 83 | 3 | | 72 | | 3 | 54 | | 347 | |
| 12 | Fruit & vegetables (packaged salads) | 54.1 | 0.7 | 6 | 31 | 6 | 32 | | 5 | 21 | 5 | | 25 | | 5 | 18 | | 127 | |
| 13 | Meat & meat products | 43.6 | 0.6 | 1 | 76 | 1 | 76 | | 1 | 89 | 1 | | 85 | | 1 | 76 | | 402 | |
| 14 | Cereal & cereal products, fruit & vegetables (nuts & dried fruit) | 39.5 | 0.5 | 7 | 128 | 7 | 178 | | 6 | 140 | 6 | | 131 | | 5 | 119 | | 696 | |
| 15 | Cereal & cereal products, fruit & vegetables (dried fruit) | 37.8 | 0.5 | 1 | 67 | 1 | 59 | | 1 | 66 | 1 | | 67 | | 1 | 85 | | 344 | |
| 16 | Confectionery | 32.8 | 0.5 | 4 | 25 | 3 | 23 | | 3 | 30 | 3 | | 38 | | 4 | 42 | | 158 | |
| 17 | Fruit & vegetables (canned), sauces & spreads | 27.4 | 0.4 | 7 | 148 | 7 | 145 | | 6 | 143 | 6 | | 143 | | 7 | 118 | | 697 | |
| 18 | Meat alternatives, sauces & spreads | 27.1 | 0.4 | 7 | 70 | 9 | 85 | | 8 | 94 | 11 | | 93 | | 6 | 81 | | 423 | |
| 19 | Meat and meat products | 22.1 | 0.3 | 1 | 39 | 1 | 34 | | 1 | 38 | 1 | | 39 | | 1 | 34 | | 184 | |
| 20 | Meat and meat products | 21.3 | 0.3 | 2 | 28 | 2 | 29 | | 2 | 39 | 2 | | 32 | | 2 | 39 | | 167 | |
| 21 | Convenience foods (soup) | 12.0 | 0.2 | 1 | 19 | 1 | 19 | | 1 | 14 | 1 | | 14 | | 1 | 11 | | 77 | |
| 22 | Dairy (ice-cream) | 9.6 | 0.1 | 3 | 37 | 3 | 35 | | 3 | 33 | 3 | | 35 | | 3 | 44 | | 184 | |
| 23 | Cereal & cereal products | 8.4 | 0.1 | 2 | 36 | 2 | 25 | | 2 | 17 | 2 | | 18 | | 2 | 19 | | 115 | |
| 24 | Dairy (yoghurt) | 8.2 | 0.1 | 2 | 36 | 2 | 39 | | 2 | 45 | 2 | | 44 | | 1 | 50 | | 214 | |
| 25 | Dairy (ice cream) | 7.0 | 0.1 | 6 | 23 | 5 | 20 | | 4 | 15 | 4 | | 25 | | 4 | 15 | | 98 | |
| 26 | Processed meat | 18.8 | 0.3 | 3 | 29 | 2 | 40 | | 2 | 30 | 2 | | 40 | | 2 | 29 | | 168 | |
| 27 | Biscuits, Cereal bars | 18.4 | 0.3 | 3 | 15 | 4 | 30 | | 3 | 32 | 3 | | 35 | | 4 | 35 | | 147 | |
| 28 | Dairy | 17.8 | 0.2 | 2 | 11 | 2 | 26 | | 2 | 39 | 2 | | 32 | | 2 | 36 | | 144 | |
| 29 | Bread & bakery | 15.5 | 0.2 | 1 | 11 | 1 | 7 | | 1 | 13 | 1 | | 14 | | 1 | 17 | | 62 | |
| 30 | Cereal & cereal products canned vegetables | 12.7 | 0.2 | 2 | 160 | 2 | 187 | | 2 | 217 | 2 | | 219 | | 3 | 208 | | 991 | |
| 31 | Bread & bakery | 8.8 | 0.1 | 7 | 85 | 6 | 85 | | 6 | 82 | 6 | | 84 | | 6 | 87 | | 423 | |
| 32 | Fish & seafood products | 8.3 | 0.1 | 2 | 23 | 2 | 24 | | 2 | 22 | 2 | | 25 | | 2 | 25 | | 119 | |
| Category: Non-alcoholic beverages | |  |  |  |  |  |  | |  |  |  | |  | |  |  | |  | |
| 33 | Soft and other non-alcoholic drinks | 273.3 | 41.1 | 26 | 173 | 26 | 221 | | 21 | 220 | 21 | | 225 | | 22 | 245 | | 1084 | |
| 34 | Soft and other non-alcoholic drinks | 184.9 | 27.8 | 18 | 160 | 21 | 198 | | 22 | 200 | 20 | | 171 | | 17 | 186 | | 915 | |
| 35 | Soft and other non-alcoholic drinks | 19.7 | 3.0 | 4 | 66 | 4 | 74 | | 5 | 70 | 5 | | 68 | | 5 | 57 | | 335 | |
| Total |  |  |  | 206 | 3469 | 217 | 3679 | | 209 | 3646 | 200 | | 3536 | | 188 | 3509 | | 17839 | |

^a^Converted from AUD to US dollars in Nov 2022 (1 AUD is 0.67 USD) AUD Australian dollars
